# Genetic variations in *GBA1* and *LRRK2* genes: Biochemical and clinical consequences in Parkinson disease

**DOI:** 10.3389/fneur.2022.971252

**Published:** 2022-08-12

**Authors:** Laura J. Smith, Chiao-Yin Lee, Elisa Menozzi, Anthony H. V. Schapira

**Affiliations:** ^1^Department of Clinical and Movement Neurosciences, Queen Square Institute of Neurology, University College London (UCL), London, United Kingdom; ^2^Aligning Science Across Parkinson's (ASAP) Collaborative Research Network, Chevy Chase, MD, United States

**Keywords:** Parkinson's disease, glucocerebrosidase, *LRRK2*, *GBA1*, lysosome

## Abstract

Variants in the *GBA1* and *LRRK2* genes are the most common genetic risk factors associated with Parkinson disease (PD). Both genes are associated with lysosomal and autophagic pathways, with the *GBA1* gene encoding for the lysosomal enzyme, glucocerebrosidase (GCase) and the *LRRK2* gene encoding for the leucine-rich repeat kinase 2 enzyme. *GBA1*-associated PD is characterized by earlier age at onset and more severe non-motor symptoms compared to sporadic PD. Mutations in the *GBA1* gene can be stratified into severe, mild and risk variants depending on the clinical presentation of disease. Both a loss- and gain- of function hypothesis has been proposed for *GBA1* variants and the functional consequences associated with each variant is often linked to mutation severity. On the other hand, *LRRK2*-associated PD is similar to sporadic PD, but with a more benign disease course. Mutations in the *LRRK2* gene occur in several structural domains and affect phosphorylation of GTPases. Biochemical studies suggest a possible convergence of *GBA1* and *LRRK2* pathways, with double mutant carriers showing a milder phenotype compared to *GBA1*-associated PD. This review compares *GBA1* and *LRRK2*-associated PD, and highlights possible genotype-phenotype associations for *GBA1* and *LRRK2* separately, based on biochemical consequences of single variants.

## Introduction

Parkinson disease (PD) is the second most common neurodegenerative disorder. The disease is characterized by the progressive loss of dopaminergic neurons in the *substantia nigra pars compacta* (SNpc) and the presence of intracellular proteinaceous inclusions, named Lewy bodies which are made up primarily of alpha-synuclein protein aggregates (1, 2). PD patients exhibit a classic triad of motor symptoms including bradykinesia, rigidity and resting tremor. A spectrum of non-motor symptoms has also been described, including cognitive decline, sleep disturbances, hyposmia and psychiatric symptoms (3).

Approximately 10–15% of all PD is caused by an identifiable genetic mutation (4), with large genome wide association studies (GWAS) having identified several additional genes and genetic loci important in familial and sporadic PD, many of which are associated with lysosomal and autophagic functions. Among these are the *GBA1* gene (OMIM 606463), which encodes the lysosomal hydrolase enzyme glucocerebrosidase (GCase; EC 3.2.1.45), and *LRRK2* (OMIM 609007) which encodes the leucine-rich repeat kinase 2 enzyme. Variants in these genes are widely recognized as the two most common genetic risk factors of PD worldwide (5–7).

In this review, we highlight the differences between *GBA1* and *LRRK2* variants, from both a clinical and biochemical perspective, and disentangle the complexity and heterogeneity of *GBA1*- and *LRRK2*-associated PD. We also summarize the recent findings on PD patients carrying both *GBA1* and *LRRK2* variants, and their particular clinical phenotype compared to single respective mutants, and possible pathomechanisms involved. Understanding the functional consequences of these variants and how they ultimately lead to specific PD phenotypes, is crucial to develop novel, gene-targeting therapies and direct patients to appropriate clinical trials.

## The *GBA1* gene to protein

**Figure 1:**
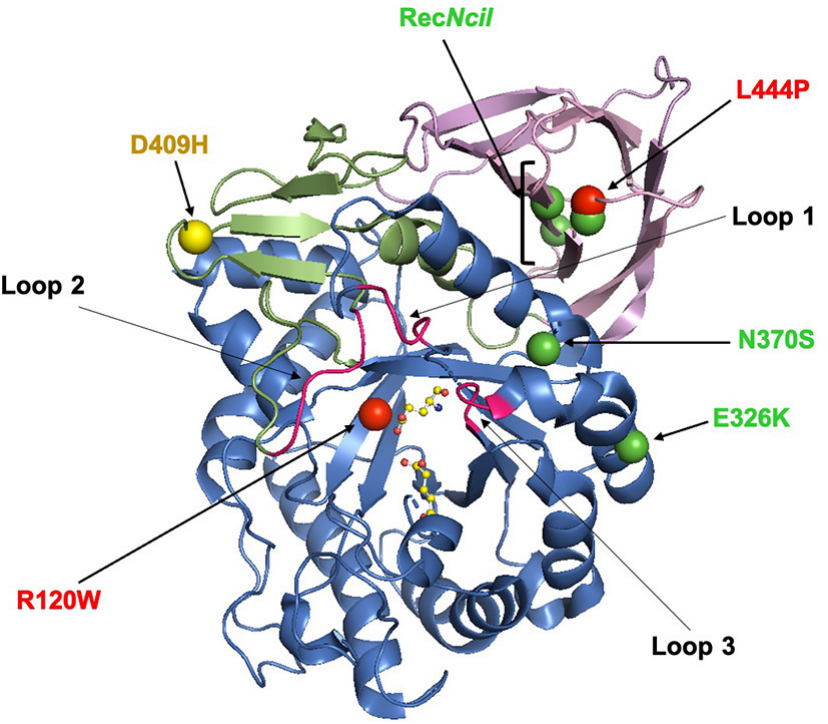
The X-ray structure of glucocerebrosidase (PDB code 3GXI). Domain I is shown in orange. Domain II is shown in pink. Domain III, the catalytic domain, is shown in blue and contains the active-site residues E253 and E340 which are shown as ball-and-stick models. The six significant glucocerebrosidase variants (R120W, L444P, E326K, N370S, D409H, and Rec*Ncil*) are shown with spheres. The color of the spheres corresponds with the odds ratio associated with the variant: green (<5); yellow (5–10) and red (>9) (13, 14). This figure was created using The PyMOL Molecular Graphics System, Version 2.0 Schrödinger, LLC.

The *GBA1* gene and is located on chromosome 1 (1q21) and is made up of 11 exons and 10 introns spanning a sequence of 7.6 kb. It encodes a 60 kDa lysosomal hydrolase enzyme, glucocerebrosidase (GCase). The mature GCase peptide consists of 497 residues and is comprised of three non-continuous domains (as shown in Figure 1). The active site is located in Domain III, which is a (β/α)_8_ triosephosphate isomerase (TIM). Domain I consists of an antiparallel β-sheet, and Domain II resembles an immunoglobulin fold made up of 8 β-sheets (8–10). Within the mature GCase structure are three important flexible loops, which cap the active site. In an acidic environment, the conformation of loop 3 changes to allow substrates to access the active site (11, 12).

GCase cleaves the sphingolipid glucosylceramide (GlcCer) into glucose and ceramide at the lysosome. Bi-allelic *GBA1* mutations cause the lysosomal storage disorder Gaucher disease (GD), which presents as widespread accumulation of GlcCer and glucosylsphingosine (GlcSph) within the lysosomes of many cell types, particularly macrophages, across several tissues and organs. GCase is folded in the endoplasmic reticulum (ER) and binds to the lysosomal integral membrane protein type 2 (LIMP-2) to be trafficked to the lysosome, through the secretory pathway where it undergoes *N*-linked glycosylation (15–17). These post-translational modifications are thought to be imperative to the production of a fully active enzyme (18).

## Common *GBA1* variants

Almost 300 unique variants have been reported in the *GBA1* gene, which span the entire protein (Figure 1). These include missense or non-sense mutations, insertions or deletions, complex alleles and splice junction mutations. The point mutations c.1226A>G (N370S) and c.1448T>C (L444P) are the most commonly associated with GD (19, 20). Generally the L444P variant causes a severe, neuronopathic type II or III GD, whereas the N370S variant is generally associated with non-neuronopathic type I GD (21). Some *GBA1* mutations arise from recombination events between the functional *GBA1* gene and a highly homologous pseudogene (*GBA1*P), an example of which is the complex allele Rec*Ncil* (19, 20).

Many mutations in the *GBA1* gene, including the common R120W variant, occur in and around the active site, influencing its stability and affecting enzyme activity. Other common mutations including, D409H and L444P, occur far from the active site, suggesting important roles for Domains I and II (9). In the case of the L444P variant, the substitution of leucine to proline causes rigidity in the protein backbone, potentially disrupting the hydrophobicity of the domain (22) which may influence protein folding. This variant is also thought to be influenced by a lack of N-linked glycosylation and subsequent structural instability (23). To date, the crystal structure of N370S *GBA1* is the only X-ray structure resolved. The N370S mutation occurs at the interface of domains II and III (9) and prevents stabilization of loop 3 at an acidic pH, impairing the ability of GCase to bind its substrate (12, 24).

## *GBA1* variants and Parkinson disease

Biallelic or monoallelic variants in the *GBA1* gene are found in 10-15% of PD cases worldwide, and up to 30% of cases of Ashkenazi Jewish (AJ) ancestry (25). The penetrance of *GBA1* variants in PD is variable. The probability of developing PD is ~5–7 and 9–12% among GD patients and 1.5–14 and 8–19% among *GBA1* heterozygous carriers, by age 60 and 80, respectively (3, 26–29).

Within PD, *GBA1* gene variants are stratified into complex, severe, mild and risk variants. The severity of a *GBA1* mutation is based upon the phenotype it presents when homozygous in those with GD. Risk variants are referred to as such as they do not present any clinical features of GD when homozygous or compound heterozygous, but increase the risk of PD (30–32).

The type of variant differently influences the risk of PD, with higher odd ratios (OR) for complex or severe variants (e.g., L444P), followed by mild (e.g., N370S) and risk (e.g., E326K) variants (OR: 15, 4, and 2, respectively) (13, 14, 30, 33), as highlighted in Figure 1.

## *GBA1-*Parkinson disease: Clinical picture and genotype-phenotype associations

From a pathological point of view, *GBA1* associated PD (*GBA1*-PD) cases present with diffuse Lewy body pathology (34–39). From a clinical perspective, the most striking differences between *GBA1*-PD and sporadic PD cases are an earlier presentation and more severe non-motor phenotype, mainly within the cognitive, psychiatric, and olfactory domains (34, 40–44). However, this more severe phenotype is more clearly recognizable in patients carrying complex or severe variants, supporting a genotype-phenotype association (42, 45).

In terms of cognitive function, *GBA1*-PD patients with mild or risk variants showed slower occurrence of cognitive impairment compared to complex or severe variants (42, 45, 46), or to non-carriers (47). Psychiatric symptoms, hallucinations and hyposmia are also more common in *GBA1*-PD vs. non-carriers (40, 42, 44, 48), and these are more frequent in carriers of severe and complex variants compared to mild or risk variants (42, 49).

Controversy surrounds disease progression in *GBA1*-PD. In one study, *GBA1*-PD was characterized by a more aggressive progression and reduced survival rates compared to non-carriers (41), however, in another longitudinal study evaluating AJ patients, no significant effect on survival of either severe or mild variants was detected (50). When stratifying by variant type, risk variants were associated with similar mortality rates compared to non-carriers (51), with the greatest association with increased mortality in patients carrying severe variants (46).

Severe variants are generally associated with faster development of motor complications (42, 51). However, more recent longitudinal studies suggest that *GBA1* status does not influence the risk of developing motor complications, even where different types of variants were considered separately (52–54).

Evaluating the biochemical consequences of *GBA1* variants and their relationship with clinical features may aid in understanding of the complexity of *GBA1*-PD. Among markers of *GBA1* dysfunction, GCase enzymatic activity is the most studied. GCase activity was found to be reduced in leucocytes (42), dried blood spots (55–57), and cerebrospinal fluid (CSF) (58) of patients with *GBA1*-PD compared to non-carriers. GCase activity presented a steeper decline among *GBA1*-PD patients according to variant severity (42). In a longitudinal analysis, increasing severity of *GBA1* variants was associated with increasingly steeper decline in GCase activity, however the latter was not correlated overall with increasing severity of motor or cognitive features (56). Similarly, no genotype-phenotype correlation was found between GCase enzymatic activity and disease severity outcomes in a cross-sectional study (57), suggesting that GCase enzymatic activity might not be a reliable marker of disease severity or progression in *GBA1*-PD. Longitudinal studies evaluating other biochemical consequences of *GBA1* dysfunction (e.g., sphingolipid metabolism), maybe in combination with GCase deficiency, and their ultimate impact on disease course, are needed.

## *GBA1* variants and Parkinson disease: Pathogenic mechanisms

Both loss- and gain- of function pathways are proposed to influence PD risk and onset (59, 60), and it is thought that these two hypotheses are not mutually exclusive. An overview of the pathogenic mechanisms associated with individual *GBA1* mutations can be found in Table 1.

**Table 1:** Overview of the pathogenic mechanisms associated with the most common *GBA1* variants associated with PD (L444P, N370S, and E326K).

	**Variant Severity**	**GCase Activity**	**ALP function**	**Lipid homeostasis**	**ER stress**	**Alpha-synuclein pathology**	**Mitochondrial function**
L444P	Severe	↓↓↓	↓	↓	↑↑↑	↑	↓
N370S	Mild	↓	↓	↓	↑	↑	↓
E326K	Risk	↓ to a lesser extent	–	↓	–	↑	–

(↓) denotes reduction in function, (↑) denotes an increase and (–) denotes unchanged or no literature surrounding this mechanism.

ALP, autophagy lysosomal pathway; ER, endoplasmic reticulum.

Variants in the *GBA1* gene often lead to a loss of GCase function. Analysis of GCase activity in the blood of PD patients has demonstrated that patients with severe *GBA1* mutations exhibit a greater reduction in GCase activity when compared to those with mild *GBA1* mutations and risk variants (56). This is supported by functional analysis of recombinant GCase protein, showing that risk variants reduce GCase activity to a lesser extent than GD-causing variants. The L444P and N370S variants reduce catalytic activity by 75–97 and 65–97%, respectively, whereas the E326K variant was associated with a 43–58% reduction (10, 61–63). The same pattern has been observed in fibroblast lines from patients harboring these mutations (64). However, this genotype-phenotype correlation is absent in one study in induced pluripotent stem cell (iPSC)-derived dopamine neurons where GCase activity was similarly reduced in L444P and N370S variants (65).

A loss of GCase activity may explain some of the downstream pathogenic mechanisms observed in models of *GBA1* variants, as in human cells a GCase deficiency was associated with lysosomal dysfunction and alpha-synuclein pathology (66). In iPSC-derived midbrain dopamine neurons, the N370S variant has been associated with a significant reduction in GCase activity and protein, accompanied by impairment of the lysosome, altered distribution of GlcCer and increased extracellular release of alpha-synuclein (67). Similarly, in neural crest stem cell-derived midbrain dopamine neurons, heterozygous N370S mutations cause a loss of GCase function, impaired macroautophagy and alpha-synuclein pathology. This was rescued by the small molecular chaperone, ambroxol, suggesting these arose due to improper trafficking and activity of N370S GCase protein (68).

In cells harboring the L444P mutation, impaired lysosomal and autophagic function has been demonstrated, accompanied by a significant reduction in GCase activity and protein (69–71). However, contrary to the hypothesis that a loss of GCase function is imperative for cellular pathology, in iPSC-derived dopamine neurons from patients with homozygous and heterozygous L444P and N370S mutations, activity did not correlate with pathology. In homozygous lines, GCase activity was reduced to a greater extent than heterozygous lines, however no difference was observed in alpha-synuclein pathology and autophagic defects (65).

Improper function of the autophagy-lysosomal pathway (ALP) can lead to the aberrant metabolism of alpha-synuclein. Such has been shown in models of L444P and N370S variants (67, 68). In L444P heterozygous mice, a significant loss of GCase activity led to an abundance of alpha-synuclein inclusions in the brain and altered levels of GlcSph (72). This variant has also been associated with increased neuronal vulnerability to and accelerated spread of alpha-synuclein pathology in mice (73, 74).

It has also been proposed that there may be a genotype-phenotype correlation between severe and mild *GBA1* variants and alpha-synuclein pathology. In SH-SY5Y cells, the L444P variant was associated with a greater increase in alpha-synuclein accumulation and stabilization, compared to N370S and wild-type (75). Another study in fibroblasts and SH-SY5Y cells demonstrated that both L444P and N370S fibroblasts exhibited an increase in the release of extracellular vesicles compared to control lines. However, alpha-synuclein pathology in SH-SY5Y cells was only promoted when incubated with vesicles isolated from L444P lines, and not N370S lines (76). In addition, a recent study showed that the E326K and L444P variants, despite different GCase activity, both presented comparable levels of alpha-synuclein aggregates suggesting that loss of GCase activity is not the only mechanism involved in alpha-synuclein pathology and that other mechanisms are involved in this process, especially for risk variants (64).

In addition to alpha-synuclein, the metabolism of lipids can be affected by impairment of the ALP or mitochondria, the latter of which has also been demonstrated in models of L444P (70, 71) and N370S (77) variants. Changes in the composition of glycosphingolipids has been demonstrated in models of *GBA1* variants, likely due to a loss of GCase function and poor lysosomal and autophagic degradation. In mice with N370S and L444P variants, a reduction in GCase function was concurrent with an accumulation of GlcSph, which promoted alpha-synuclein aggregation (78). Similarly, in N370S iPSC-derived dopamine neurons an accumulation of GlcCer and alpha-synuclein was observed (79).

Accumulation of glycosphingolipids may be key to the pathology of L444P and N370S *GBA1* variants as in dopamine neurons with these variants, reducing the levels of GlcCer/GlcSph rescued alpha-synuclein pathology (79, 80). Interestingly, in one study of L444P mice an accumulation of GlcSph alone was observed, which accelerated alpha-synuclein aggregation (72). In fibroblasts from L444P heterozygous patients, a significant increase in glycosphingolipids has been demonstrated, which correlated with decreased GCase activity. When these lipids were extracted and incubated with recombinant alpha-synuclein, an increase in the pathogenic aggregation of alpha-synuclein was observed, due to a higher content of short-chain lipids in the L444P cells (81). This may occur as lipid membrane dynamics are required for macroautophagy and chaperone mediated autophagy (CMA) (82).

In addition to glycosphingolipids, the level of fatty acids may be altered by *GBA1* variants. In SH-SY5Y cells, expression of the E326K variant led to increased accumulation and formation of lipid droplets, which was accompanied by alpha-synuclein aggregation (64), suggesting alterations in the metabolism of several lipid types may be key to *GBA1* pathology.

An additional pathogenic mechanism that has been proposed for *GBA1*-associated PD arises from the toxic gain-of-function hypothesis. As the majority of *GBA1* variants are missense, a misfolded protein is often produced and retained in the endoplasmic reticulum (ER). This can activate ERAD, and lead to a deficiency in enzyme level through degradation and can activate pathway such as the unfolded protein response (UPR) and eventual ER stress. In some studies, in fibroblasts and Drosophila, activation of the UPR has been demonstrated in L444P and N370S variants (83, 84). Conversely, other studies have suggested a genotype-phenotype correlation between variant severity of UPR activation. In fibroblasts and SH-SY5Y cells, L444P has been associated with ER retention and ER stress, which was absent in N370S and E326K cells (64, 84). In another study, the severe L444P variant displayed extensive ERAD (85), suggesting that the extent of ER stress may correlate with disease severity, perhaps due to more pronounced conformational changes. However, another fibroblast study has demonstrated heterogeneity in ER retention and degradation across lines with the N370S genotype (86), weakening the genotype-phenotype correlation argument.

Overall, current evidence suggests that the mechanisms in which *GBA1* variants predispose to PD are multifaceted. Different pathogenic mechanisms could explain the differences in risk and phenotypes of PD for single variants, and future studies will need to address these questions. The reasons why the majority of GD patients or heterozygous carriers do not develop PD, also remain unexplained.

## *GBA1-*Parkinson disease: Current and future therapeutic strategies

The discovery of the *GBA1* gene in PD has opened a new avenue to develop novel therapeutics for PD, with several *GBA1*-targeted strategies under development with the aim to enhance GCase activity [reviewed in smith et al. (87)].

Significant focus is on the development of molecular chaperones to penetrate the blood-brain-barrier (BBB) to bind and refold GCase in the ER, facilitating trafficking and rescuing enzyme activity (88). Within this class is the inhibitory, pH-dependant small molecular chaperone, ambroxol (89), which has been shown to increase GCase activity and reduce alpha-synuclein pathology in several cell and animal models (68, 90–95). Ambroxol has also demonstrated the ability to reduce UPR activation in *Drosophila* models of GCase deficiency (84, 96). In Type 1 GD patients, ambroxol has been shown to be safe and tolerable (ClinicalTrials.gov Identifier: NCT03950050) (97) and results from a phase II, single-centre trial, in PD patients with and without *GBA1* mutations, demonstrate that ambroxol can cross the BBB and enter the CSF where it can alter GCase activity and protein level (ClinicalTrials.gov Identifier: NCT02941822) (98). Ambroxol also increased the alpha-synuclein concentration in the CSF and, importantly, improved motor function. A phase III clinical trial of ambroxol in treating PD is expected to commence in early 2023.

In addition to inhibitory chaperones, development of non-inhibitory chaperones for GCase is underway. Two compounds, NCGC758 and NCGC607, have been shown to improve GCase trafficking and rescue glycosphingolipid and alpha-synuclein accumulation in iPSC-derived dopamine neurons from *GBA1*-PD patients (99, 100).

Allosteric modulator small molecules, that can bind and enhance GCase activity, are also an area of interest. An example of which is LT1-291, which has been shown to cross the BBB (Trialregister.nl ID: NTR7299) (101). Pre-clinical studies have demonstrated that LT1-291 can reduce substrate accumulation (101), and this was also shown in a phase 1b placebo-controlled trial in *GBA1*-PD patients (NL6574). Further clinical trials are expected.

Small molecules are also being developed to modulate GCase activity through targeting other proteins. An example of this are histone deacetylase inhibitors (HDACis), which have been shown to increase GCase activity by preventing its ubiquitination and degradation (102, 103) or improving GCase folding and trafficking (104) in GD fibroblasts.

Enzyme replacement therapy (ERT) has shown great efficacy in improving the visceral symptoms of GD but fails to cross the BBB (105). Currently research is underway to improve the delivery of wild-type GCase enzyme and enhance its ability to cross the BBB. This involves ligating a peptide, usually a virus-associated protein, to the GCase enzyme (106). Denali Therapeutics have recently developed the transport-vehicle-modified recombinant GCase enzyme (ETV:*GBA1*) compound, using their transport vehicle platform technology which has the potential to actively transport enzymes across the BBB (107). Preclinical research is underway with this compound, but further studies are needed to investigate its efficacy in *GBA1*-PD patients.

Another avenue being explored to deliver wild-type GCase enzyme to the brain is gene therapy. Most commonly, the *GBA1* gene is ligated into the adeno-associated virus (AAV) vector, and delivered to the brain. In mouse models of GD this method has been shown to rescue GCase activity and expression, reduce alpha-synuclein pathology and decrease glycosphingolipid accumulation (108–111). Prevail Therapeutics are currently testing their PR001A compound, which delivers the *GBA1* gene using the AAV-9 vector, in phase I clinical trials (ClinicalTrials.gov Identifier: NCT04127578 and NCT04411654).

Strategies targeted to GCase to reduce the accumulation of glycosphingolipid substrates are also under development. Substrate reduction therapy (SRT), miglustat, has shown efficacy in reducing lipid accumulation in dopamine neurons from PD patients with *GBA1* mutations, and can reduce alpha-synuclein pathology when coupled with GCase over-expression (79). However, miglustat cannot cross the BBB. Novel brain penetrant SRTs are therefore being developed. Sanofi's venglustat (GZ667161) had shown promise in GCase-deficient synucleinopathy mice models, able to reduce alpha-synuclein and glycosphingolipid accumulation and improve cognitive function (112). The phase I trials of venglustat demonstrated successful target engagement (ClinicalTrials.gov Identifier: NCT01674036 and NCT01710826), however the phase II trial failed to show a benefit, with patients with *GBA1* mutations exhibiting a decline in motor function in PD (ClinicalTrials.gov Identifier: NCT02906020).

## The *LRRK2* gene to protein

**Figure 2:**
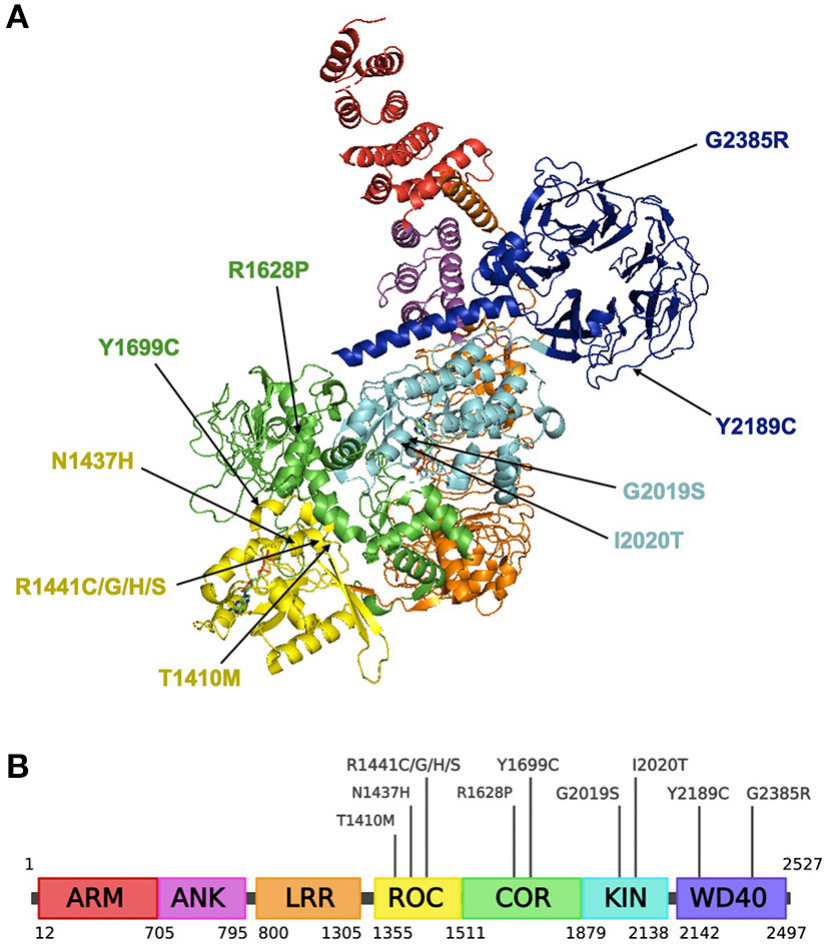
Structure of the *LRRK2* protein and residing pathogenic variants. **(A)** Structural ribbon model of the *LRRK2* monomer. PDB: 7LHW. This figure was created using The PyMOL Molecular Graphics System, Version 2.0 Schrödinger, LLC. **(B)** Full-length *LRRK2* protein. Created with BioRender.com.

The *LRRK2* gene (also known as *PARK8*), first discovered in 2002 encodes for the leucine-rich repeat kinase 2 (*LRRK2*, OMIM 609007) (113). It is located on chromosome 12, consists of 51 exons and encodes a large, 288 kDa multi-domain protein containing seven domains (as illustrated in Figures 2A,B): armadillo repeat motif (ARM); ankyrin repeat (ANK); leucine-rich repeat (LRR); Ras of complex (ROC) GTPase domain; C-terminal of ROC (COR) domain; kinase (KIN) domain; WD40 domain (114). *LRRK2* is thought to dimerize *via* the ROC-COR and WD40 domains, while the WD40 domain has also been implicated in *LRRK2*-mediated neurotoxicity (115–117).

*LRRK2* is expressed ubiquitously in the brain, including neurons and glial cells, as well as in the kidneys, lungs, liver, heart and immune cells (118–121). The *LRRK2* protein is thought to be primarily cytosolic but can also localize to a subset of organelles and inner cellular membranes, including mitochondria, ER, Golgi apparatus and microtubules (122, 123). However, the physiological roles of *LRRK2* remain unclear, although it is suggested to be involved in many different processes such as adult neurogenesis, scaffolding, homeostasis of lysosome-related organelles, the innate immune response and neuroinflammation (124–126).

## Common *LRRK2* variants

There are several LRRK2 missense variants that have been confirmed to increase PD risk, including the most common variant G2019S, as well as N1437H, R1441C/G/H/S, Y1699C and I2020T (127, 128).

As seen in Figures 2A,B, G2019S resides in the activation loop of *LRRK2*'s ATP binding site which regulates *LRRK2* kinase activity (129). A computational prediction study suggests that G2019S may decrease the flexibility of the loop and improves the stability of the kinase domain, enabling it to remain in an active conformation for an extended period (130). This has been shown to increase phosphorylation of substrates by 2- to 3-fold (131). Another variant associated with increased PD risk, I2020T, is also located in the activation loop of the kinase domain and has been reported to significantly increase *LRRK2* autophosphorylation by around 40% relative to the native enzyme (122).

Other variants that do not reside in the kinase domain may also modify *LRRK2* kinase activity. The ROC domain contains motifs that are conserved amongst GTP-binding proteins, suggesting that *LRRK2* is a functioning GTPase that can regulate *LRRK2* kinase activity (132–134). An *in vitro* study showed that the R144C/G/H/S mutations located in the ROC domain, increases kinase activity while decreasing GTP hydrolysis and weakening LRRK2 dimerisation (132). N1437H in the ROC domain has been proposed to impair monomer-dimer conformational dynamics and hinder GTPase activity, permanently locking LRRK2 into a dimeric state (135). T1410M, found in the ROC domain, is a novel variant with unclear pathogenicity and may distort the tertiary structure of LRRK2 and disrupt GTP hydrolysis (136). Meanwhile, the Y1699C variant resides in the COR domain and is proposed to strengthen ROC-COR interactions, weaken ROC-COR dimerization and reduce GTPase activity (137).

Y2189C, identified in Arab-Berber populations (138), is located within the WD40 domain is presumed to have a deleterious effect for *LRRK2* and induces high levels of cellular toxicity (139), however there is still controversy surrounding its pathogenicity for PD (128, 138). The G2385R and R1628P variants act as potential genetic risk factors in Chinese and Malaysian populations (140–142). G2385R is also located within the WD40 domain and causes dysfunctional synaptic vesicle trafficking (128, 143, 144), while R1628P is located in the COR domain.

## *LRRK2* gene variants and Parkinson disease

Worldwide, the frequency of *LRRK2* G2019S is found in 1% of sporadic PD and 4% of familial PD cases (145). It is most frequently found in sporadic PD cases of north African Arabs and of AJ descent (30 and 10% of cases, respectively), whereas the variant is rarely found in Asians (only 0.1%) (145).

The penetrance of PD in subjects carrying a *LRRK2* mutation is not fully elucidated and varies with age, which may explain both the high prevalence of mutations in sporadic PD cases and the detection of mutations in unaffected individuals (145). Although this finding has been repeatedly reported, the precise mutation penetrance rates vary across studies due to different populations considered and methodologies applied, and it is unclear whether distinct variants can differently impact on penetrance. Overall, cumulative risk has been estimated to be around 30–40% at age 80, with variable figures ranging from 7 and 80% (145–150). In one study considering effects of pathogenic *LRRK2* mutations on penetrance, carriers of G2019S showed a lower penetrance compared to carriers of other pathogenic mutations combined, although the group of non-G2019S was relatively small (145).

## *LRRK2*-Parkinson disease: Clinical picture and genotype-phenotype associations

*LRRK2*-PD patients are clinically very similar to sporadic PD. There are no differences in age at onset between *LRRK2*-PD patients carrying pathogenic variants vs. non-carriers (151, 152), as well as between carriers of different pathogenic mutations (G2019S vs. R1441C/G/H) (127), or carriers of risk variants vs. non-carriers (141) or vs. carriers of pathogenic variants (153). Interestingly, the male predominance seen in PD is less represented within *LRRK2*-PD patients (151, 152).

The motor phenotype of *LRRK2*-PD is that of levodopa-responsive parkinsonism, with sustained response over time, later onset of levodopa-induced dyskinesia (145, 151), and milder progression in motor symptoms over time (152) compared to non-carriers.

Although data comparing different genotypes is limited, there may be genotype-phenotype associations within *LRRK2*-PD, with risk variants showing a more rapid progression and G2019S a more benign course. Higher incidence of postural instability gait difficulty (PIGD) sub-type has been reported in PD patients of both AJ origin carrying G2019S (151, 152, 154), and Chinese origin carrying G2385R (155), when compared to non-carriers. Similar rates of PIGD sub-type were found in G2019S and G2385R when compared together (156). Within pathogenic variants, PD patients with G2019S showed more frequent PIGD when compared to patients carrying the R1441G variant (127). When analyzing disease course, carriers of pathogenic variants showed more sustained response to levodopa and lower motor scores when compared to carriers of risk variants (153, 156), and survival curves of AJ G2019S PD carriers were also not different from those of non-carriers (50, 157). Within pathogenic mutations, motor fluctuations were more frequently reported in carriers of p.R1441C/G/H mutation than in carriers of p.G2019S mutation (127).

From a non-motor perspective, the phenotype of all *LRRK2*-PD patients seems to be more benign compared to that of non-carriers. Slower cognitive decline has been observed in *LRRK2*-PD compared to sporadic PD or *GBA1*-PD (145, 158). Carriers of G2019S PD patients also showed better olfactory function, less severe mood disorders, and less frequent REM sleep behavior disorders (RBD) (159, 160) compared to non-carriers (156). In a cohort of Chinese patients, carriers of G2385R presented better cognitive performances and more severe RBD symptoms compared to non-carriers (155).

Overall, a genotype-phenotype relationship among *LRRK2*-PD patients might exist, with pathogenic variants showing a more benign motor disease course compared to risk variants. These observed clinical differences could reflect a lower pathogenicity for p.G2019S mutation, however additional genetic and environmental factors beyond mutational status might contribute to these different manifestations.

## *LRRK2* gene variants and Parkinson disease: Pathogenic mechanisms

As a major player in the ALP, pathogenic *LRRK2* mutations have been shown to alter lysosomal activity, including late stage endocytosis, lysosome trafficking and synaptic vesicle endocytosis (161). In primary mouse astrocytes, G2019S, R1441C, and Y1699C reduce lysosomal capacity and increase lysosome size, and G2019S also reduces lysosomal pH, which is associated with dysfunctional lysosomal activity (162). Some reports also suggest a gain-of-function mechanism for G2019S involving ER stress and UPR, although the precise mechanisms and how they may underlie PD are poorly understood (163–165).

G2019S heterozygous and homozygous mice are reported to exhibit impaired extracellular release of dopamine and profound abnormalities of mitochondria in the striatum (166). More recent studies show that G2019S knock-in mice exhibit increased dopamine transporter levels, dopamine uptake and phosphorylation of α-synuclein from 9 months of age, while *LRRK2*-KO mice show slight elevation of total α-synuclein immunoreactivity at 23 months of age (167, 168). In addition, G2019S also alter glutaminergic synaptic transmission in midbrain dopaminergic neurons of 10–12 month old (middle-aged) mice which reflects aging before the onset of motor symptoms in PD (169). In astrocyte-dopaminergic neuron co-cultures from G2019S *LRRK2*-carrying PD patients, astrocytes accumulate α-synuclein and the neurons display shortened neurites and neurodegeneration which are not seen in co-cultures with control-patient-derived astrocytes (170). Collectively, this suggests that gain-of-function *LRRK2* variants may increase the susceptibility of dopaminergic neurons to degeneration and implicates *LRRK2* in α-synuclein clearance and homeostasis in PD pathology.

Although most studies focus on gain-of-function *LRRK2* variants, large-scale genetic sequencing suggests that loss-of-function variants can also reduce *LRRK2* protein levels in around 82% of heterozygous carriers. However, loss-of-function variants may not be strongly associated with a specific PD phenotype (171). This not only further emphasizes the link between increased kinase activity and familial PD, but also highlights the importance of additional research to elucidate both the physiological functions of *LRRK2* as well as the precise mechanisms in which *LRRK2* variants influence PD risk, onset and progression.

## Rab proteins linked to *LRRK2* in Parkinson disease

*LRRK2* kinase has been shown to phosphorylate a subset of GTPases, called Rab GTPases (172). Rab proteins play important roles in vesicle trafficking, regulating the formation, transport, tethering and fusion of vesicles specific to each specific Rab, and dysfunction in Rab-mediated vesicle trafficking has been implicated in PD pathology (173). Although G2019S has been shown to increase phosphorylation of Rab proteins, *in vivo* assays show that other mutations such as R1441G also enhance Rab phosphorylation by up to 20-fold (172, 174). However, dysfunctional mutant T1348N *LRRK2* demonstrates reduced kinase activity, suggesting the importance of GTP-binding in downstream signaling events (175).

RAB29, also known as RAB7L1, is contained within the PD-linked PARK16 locus (176, 177). RAB29 is thought to be the master regulator of *LRRK2*, recruiting *LRRK2* to the *trans-*Golgi network and stimulating kinase activity. The R1441G/C and Y1699C pathogenic variants have been shown to enhance this recruitment (175), and GTP-binding is thought to be crucial for RAB29-mediated activation of *LRRK2*. This then triggers downstream phosphorylation of various Rab proteins, such as RAB8A/B and RAB10 (124, 178).

RAB8A/B and RAB10 have been shown to be involved in primary ciliogenesis, although direct links between *LRRK2* and ciliogenesis in PD have yet to be established (124). RAB29, RAB8A, and RAB10 are all implicated in maintaining lysosome homeostasis, and Liu et al. reported that phosphorylated RAB10 may also play a role in phagocytic immune response (179), further supporting any links between *LRRK2* and lysosomal dysfunction in PD (180).

## *LRRK2* gene variants and Parkinson disease: Current and future therapeutic strategies

There have been many recent developments in LRRK2-targeted strategies in PD, with a strong focus on small molecule LRRK2 kinase inhibitors which has been shown to trigger neuroprotective effects (181, 182).

The majority of LRRK2 kinase inhibitors are ATP-competitive, where the molecules compete with ATP for binding to the ATP-binding pocket in the kinase domain (183, 184). MLi-2 is a compound that exhibits exceptional potency and specificity both *in vitro* and in mouse models, where it has been shown to be well-tolerated with no adverse effects on body weight, food intake or behavior (185, 186). Although MLi-2 failed to slow or halt the progression of PD in mice and never reached clinical trials, it is an important compound for researchers to study LRRK2 function and pathobiology. PF-06685360, or PFE-360, also shows high potency, kinase selectivity and good brain permeability in rats (187). Two inhibitors [DNL-201 and DNL-151 (NCT03710707 and NCT04056689, respectively, https://clinicaltrials.gov)] are already in clinical trials (188, 189).

However, there are several challenges facing therapies targeting LRRK2 kinase. As LRRK2 protein expression is not limited to only the brain, it is crucial to assess any adverse effects on other systems in the body, such as in kidneys, lungs and immune cells. Preclinical toxicology studies show possible kidney and lung pathology as a results of various LRRK2 inhibitors (185, 187, 190, 191), and both activation and inhibition of LRRK2 kinase in immune cells have been associated with immune function (192).

Another challenge is the current lack of biomarkers and scalable assays that can measure LRRK2 activity in patients. To date, the most promising candidate biomarker is phosphorylated Rab protein (172, 193), as well as levels of auto-phosphorylated LRRK2 at Ser1292 (194). For example, phosphorylated RAB10 has been shown to be significantly increased in the brain of idiopathic PD patients (195). The development of reliable biomarkers is critical for early PD diagnosis (193), patient selection for the enrolment to clinical trials, to identify patients in which LRRK2 inhibition may be most effective and allow for personalized dose adjustments (196).

Finally, although increased LRRK2 kinase activity is present in other forms of genetic PD and especially sporadic PD (197), further research must be conducted to assess LRRK2 activity and function in these forms of PD order to assess the viability of LRRK2 inhibitors to treat all types of PD.

## *GBA1* and *LRRK2* interactions

Although there are many clear differences between *GBA1-* and *LRRK2*- associated PD, highlighted in Table 2, there is increasing evidence suggesting a possible interaction between *GBA1* and *LRRK2* in PD (193). Clinical studies show that individuals carrying both the G2019S *LRRK2* variant and a *GBA1* variant exhibit symptoms that closely mimic G2019S-*LRRK2* PD symptoms and are milder than patients carrying only a *GBA1* variant. This includes slower rates of cognitive and motor decline and milder olfactory dysfunction (158). Compound variant carriers may have higher risk of developing PD, coupled with a tendency for a slightly earlier age at onset, compared with patients carrying just one variant and sporadic PD patients (158, 198–200). This suggests that the G2019S *LRRK2* variant might be dominant over pathogenic *GBA1* variants, although it could also depend on the varying penetrance of the two genes. In addition, it is also possible that the *GBA1*/*LRRK2*-PD patients in the study are exhibiting *LRRK2*-mediated PD and the *GBA1* variants act as a bystander in pathology progression.

**Table 2:** Overview of the clinical presentation and pathological differences between *GBA1*- and LRRK2- associated PD.

	***GBA1***	***LRRK2***
Age at onset	~5 years earlier than sPD	–
Disease progression	Faster than sPD	Slower than sPD
Motor symptoms	Worse than sPD	Worse than sPD
Non-motor symptoms	Worse than sPD	Better than sPD
Cognition	Faster decline	Slower decline
Enzyme activity	Reduced	Increased
Lysosomal function	Reduced	Reduced
ALP function	Reduced	Reduced
Mitochondrial function	Reduced	Reduced
Lipid homeostasis	Reduced	–
ER stress	Increased	Increased
Rab protein phosphorylation	–	Increased
Alpha-synuclein pathology	Increased	Increased

(sPD) denotes sporadic PD; (–) denotes no change.

ALP, autophagy lysosomal pathway; ER, endoplasmic reticulum.

Biochemical studies appear to support a link between *LRRK2* kinase activity and GCase activity. For example, G2019S and R1441G/C variants reduce GCase activity (but not GCase protein levels) in dopaminergic neurons through increased RAB10 phosphorylation (200, 201). However, G2019S and the gain-of-function *LRRK2* variant M1646T [association with PD risk is unclear (138, 202)] are reported to increase GCase activity in dried blood spots (203, 204). Therefore, the influence of *LRRK2* variants on GCase activity appears to be inconsistent between the blood and dopaminergic neurons. However, there are currently a lack of studies focussing on GCase activity on *LRRK2* which warrants further investigation. In addition, progression and onset are very difficult to study in cell models and compound mutant carriers are extremely rare, posing further difficulties in investigating the convergence of the two pathways.

## Concluding remarks

The discovery of the *GBA1* and *LRRK2* mutations as the most important genetic risk factors for developing PD has led to enhanced understanding of the underlying causes of PD. Understanding the functional consequences associated with individual variants is imperative to develop highly efficacious gene-targeted therapies to halt or restore neurodegeneration. Further evaluation of *GBA1* and *LRRK2* variants and clinical presentation, as well as investigations into interactions between the two genes, is needed to develop biomarkers for early diagnosis and intervention and treatment of PD.

## Author contributions

LJS, C-YL, EM, and AHVS contributed to drafting and editing the manuscript and have read and agreed to the published version of the manuscript.

## Funding

This research was funded in part by Aligning Science Across Parkinson's (Grant number: ASAP-000420) through the Michael J. Fox Foundation for Parkinson's Research (MJFF) and by the EU Joint Programme—Neurodegenerative Research (JPND) through the MRC grant code MR/T046007/1. For the purpose of open access, the author has applied a CC BY 4.0 public copyright license to all Author Accepted Manuscripts arising from this submission. AHVS was supported by the National Institute for Health Research University College London Hospitals Biomedical Research Centre.

## Conflict of interest

The authors declare that the research was conducted in the absence of any commercial or financial relationships that could be construed as a potential conflict of interest.

## Publisher's note

All claims expressed in this article are solely those of the authors and do not necessarily represent those of their affiliated organizations, or those of the publisher, the editors and the reviewers. Any product that may be evaluated in this article, or claim that may be made by its manufacturer, is not guaranteed or endorsed by the publisher.
